# Performance of Field’s Stain Compared with Conventional Giemsa Stain for the Rapid Detection of Blood Microfilariae in Gabon

**DOI:** 10.4269/ajtmh.22-0061

**Published:** 2022-07-05

**Authors:** Franck-A. Ekoka Mbassi, Ghyslain Mombo-Ngoma, Wilfrid Ndzebe Ndoumba, Emmanuel K. Yovo, Kirsten A. Eberhardt, Dorothea Ekoka Mbassi, Ayôla A. Adegnika, Selidji T. Agnandji, Marielle K. Bouyou-Akotet, Michael Ramharter, Rella Zoleko-Manego

**Affiliations:** ^1^ Centre de Recherches Médicales de Lambaréné, Lambaréné, Gabon;; ^2^ Department of Tropical Medicine, Bernhard Nocht Institute for Tropical‚ Medicine & I. Department of Medicine, University Medical Center Hamburg-Eppendorf, Hamburg, Germany;; ^3^ Department of Parasitology-Mycology, Faculty of Medicine, University of Health Sciences, Libreville, Gabon;; ^4^ German Center for Infection Research (DZIF), Partner Sites Hamburg-Lübeck-Borstel-Riems ;; ^5^ Institute for Tropical Medicine, University of Tübingen, Tübingen, Germany

## Abstract

Filarial infections caused by *Loa loa* and *Mansonella perstans* are a considerable public health burden in rural regions of Central Africa. Rapid diagnostic tools for the detection of microfilariae in the blood are needed. Field’s stain is a rapid staining technique for microscopic slides originally established for malaria diagnostics. It requires less than 1 minute of staining compared with conventional staining protocols requiring at least 15 to 20 minutes for staining and could thus significantly accelerate diagnostics for human filariasis. Here we evaluated Field’s stain as a rapid staining technique in comparison to Giemsa stain for the detection of microfilariae in peripheral blood. Blood smears were collected from 175 participants residing in the region of Lambaréné and Fougamou, Gabon. Each participant’s samples were stained in parallel with Field’s stain and conventional Giemsa stain. Slides were then microscopically assessed and compared for qualitative and quantitative results by a blinded assessor for the two endemic filarial blood pathogens *M. perstans* and *L. loa*. Field’s stain shows excellent diagnostic performance characteristics for *L. loa* microfilariae compared with Giemsa staining. Concordance was favorable for *M. perstans* although lower than for *L. loa*. Field’s stain offers a rapid alternative to Giemsa stain for detection of *L. loa* microfilariae in thick blood smears. This could help accelerate diagnostics of blood filarial pathogens in mass screening programs or resource constrained health care institutions with high patient load.

## INTRODUCTION

*Loa loa*, the African eyeworm, *Mansonella* spp., and to a lesser degree *Onchocerca volvulus*, are human filarial pathogens endemic in Gabon.^1^^–^^3^ In 2011, the overall national prevalence was estimated at 22% for microfilaremic *L. loa*, and 10% for microfilaremic *Mansonella perstans *infections with significant variation by the respective ecosystems (forest, savannah, Ogooué river basin).^4^ Loiasis is geographically confined to Central and West Africa and is caused by a nematode transmitted by the bite of a tabanid fly of the genus *Chrysops *sp. In contrast, *M. perstans* occurs throughout the tropics and is transmitted by biting midges of the genus *Culicoides*.^5^^,^^6^

Diagnosis is established by parasitological, immunological, and molecular methods. Microscopy of peripheral blood is performed most commonly in routine care in tropical settings for the detection of microfilariae. Microscopy can be performed on fresh untreated blood samples, but staining improves the ability to discern the different filarial species based on morphology. Concentration techniques help increase sensitivity of microscopic and molecular diagnostic assays.

The thick blood smear technique that is done routinely for malaria diagnostics is among the most commonly used techniques for the detection of microfilariae in routine care in the tropics. However, its realization requires at least 30 minutes and its sensitivity is limited to approximately 60 microfilariae per milliliter (Mf/ml) similar to direct examination of fresh blood.^7^ This time-consuming method makes population-based screening programs difficult to implement.

A faster diagnostic method would therefore help for population-based screening programs. Here, we evaluate Field’s stain, a rapid version of the Romanowsky staining method originally developed to detect malaria parasites, as a potential rapid staining technique for blood microfilarial pathogens.^8^ The staining time using Field’s stain is only 1 minute; therefore significantly accelerating overall time for diagnosis. The overall objective of this work was to evaluate the performance of Field’s stain compared with conventional Giemsa stain for the detection of blood microfilariae by evaluating the diagnostic sensitivity, specificity and feasibility of species diagnosis for *L. loa *and* M. perstans* in an endemic region of Gabon.

## METHODS

### Study design and study population.

This cross-sectional study was conducted from October 31 to December 31, 2019 at the Center de Recherches Médicales de Lambaréné (CERMEL) in Gabon.^9^ Subjects of both sexes older than 2 years living in the region of Ngounié (Tsamba-Magotsi/Fougamou) and Moyen-Ogooué (Lambaréné) were invited to participate if signs and symptoms for loiasis such as history of eye worm migration, calabar swelling, and pruritus were suspected after provision of informed consent. This region is endemic to malaria, *L. loa*,* M. perstans*, and other parasitic infections. The periodicity of microfilariae of *L. loa* leads to peak microfilaremia during the daytime. Accordingly, sampling was done between 10 am and 2 pm.^10^

### Diagnostics.

#### Sampling.

For each subject living in the study area and consenting to participate in this study, a questionnaire-based interview was conducted by the investigator, and data were recorded on a case report form. Each form had a unique identifier that was used to label the microscopic slides for further assessment in the laboratory. For each subject, two thick blood smears of 10 μL of capillary blood each were performed consecutively according to the Lambaréné method.^11^ The first tick blood smear slide was labeled “A” and the second was labeled “B,” along with a subject identifier. Once the slides were air-dried, they were stored in slide trays or boxes and further transported to the laboratory and stored in appropriate long-term storage boxes. The conditions for transporting and storing of the slides were controlled to avoid any direct contact between the slides.

#### Characteristics, preparation of the reagents, and staining.

Giemsa stain was prepared using phosphate-buffered solution at pH 7.2 to obtain a final dilution of 10% Giemsa stain. Staining solutions were used fresh and no more than 4 to 5 hours after preparation. Staining time was 15 minutes before each slide was rinsed with a gentle flow of tap water until the excess dye was removed and again air-dried.

Field’s stain is a Romanowsky-based staining technique composed of two dyes: Field’s stain A is a dark purple solution containing methylene blue and azure dissolved in a phosphate buffer solution. It allows visualization of the nuclei and is the basic part of the dye. Field’s stain B is orange and contains eosin Y in a phosphate-buffered solution, allowing visualization of cytoplasma and constitutes the acid part of the dye. The products used in this study were Field’s stains A and B from Alfa Aesar (Haverhill, MA; stain A, stock number A18577, LOT 10173517; Field’s stain B, stock number A18578, LOT 10168215). Field’s stain A was prepared according to instructions mixing 5 g of commercially available powder in 600 ml of distilled water that is heated to 80°C or kept at 60°C for 30 minutes until the powder is dissolved and subsequently filtered. Field’s stain B was prepared by mixing 4.8 g of powder B in the same manner as Field’s stain A. Two jars of wide-necked bottles were filled with blue field’s stain A and red Field’s stain B. Each blood smear was immersed in Field’s B stain (red) for 5 to 6 seconds. After gently rinsing with tap water, Field’s A stain (blue) was applied for 10 to 30 seconds, followed by subsequent air-drying of the slide.

#### Light microscopy.

The lecture of the slides took place on a LEICA DM 1000 LED microscope. Study codes were masked for the microscopist for blinding of the identity of the patient. The examination was done from one end to the other and from top to bottom scanning the entire surface of the slide. Microfilariae were searched at 100× magnification. For better visibility of the morphological characteristics, species differentiation was done at 1,000× magnification.

### Statistical considerations and analysis.

The Spearman rank correlation coefficient ρ (rho) was calculated as a measure of strength of the relationship between the quantitative test results.^12^ Cohen’s κ (kappa) indicated the agreement between the qualitative results of the staining methods with the strata poor (< 0.00), slight (0.00–0.20), fair (0.21–0.40), moderate (0.41–0.60), substantial (0.61–0.80), and almost perfect (0.81–1.00), as described elsewhere.^13^ Two-sided *P* values were presented, and an α of 0.05 was determined as the cutoff for significance. All statistical analyses were performed using R (version 4.0.5, R Foundation for Statistical Computing, Vienna, Austria).

### Ethical considerations.

Each participant’s personal information was kept confidential for data acquisition and analysis. Blood sampling took place as part of an ongoing clinical trial for the treatment of loiasis (PACTR201807197019027). This study was approved by the institutional Ethics Committee of the CERMEL.

## RESULTS

### Baseline characteristics.

In the period from October 31 to December 31, 2019, 175 subjects were recruited from 23 villages. More than half of these participants resided in five villages. The sex ratio was almost even with 94:81 male-to-female ratio, and the median age was 35 years (interquartile range [IQR] 28–49 years). Most participants were between 15 and 60 years of age (88.6%); only 2% and 10%, respectively, were younger than 15 or older than 60 years.

### Microfilaria and species determination in Giemsa-stained–treated and Field’s stain–treated samples.

Almost half of all samples were positive for *L. loa* (49.7% of Giemsa-stained and 46.9% of Field’s stain treated samples). The positive rate for *M. perstans* was lower with 24.6% of Giemsa-stained and 21.1% of Field’s stain treated samples (Table 1). In statistical analysis for the presence of microfilariae of each species there was an almost perfect agreement between *L. loa* detection and a substantial agreement between *M. perstans* detection for both staining methods according to Landis and Koch (Cohen’s κ 0.92, 95% confidence interval [CI]: 0.86–0.98] for *L. loa* and 0.74, 95% CI: 0.61–0.86 for *M. perstans*).

**Table 1:** Proportion of microfilariae in Giemsa-stained and Field’s-stained samples

Species		Qualitative assessment			
		Field’s stain			
		Positives (%)	Negatives (%)	Total (%)	Cohen’s κ (95% CI)
*Loa loa*					0.92 (0.86–0.98)
Giemsa stain	Positives (%)	81 (46.29)	6 (3.42)	87 (49.71)	
	Negatives (%)	1 (0.57)	87 (49.71)	88 (50.29)
	Total (%)	82 (46.86)	93 (53.14)	175 (100.00)
*Mansonella perstans*					0.74 (0.62–0.86)
Giemsa stain	Positives (%)	32 (18.29)	11 (6.29)	43 (24.57)	
	Negatives (%)	5 (2.86)	127 (72.57)	132 (75.43)	
	Total (%)	37 (21.14)	138 (78.86)	175 (100.00)	

CI = confidence interval.

**Figure 1.**
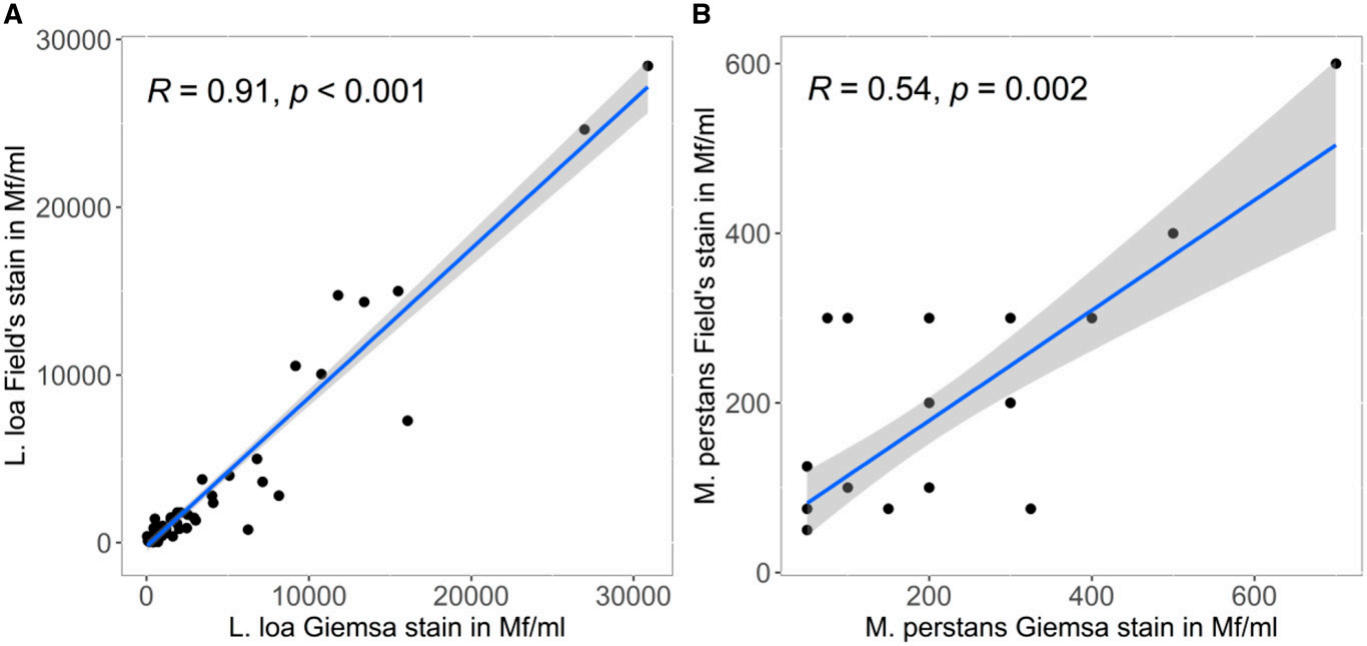
Quantitative agreement of microfilaremia quantification. *R* = Spearman’s ρ. This figure appears in color at www.ajtmh.org.

**Figure 2.**
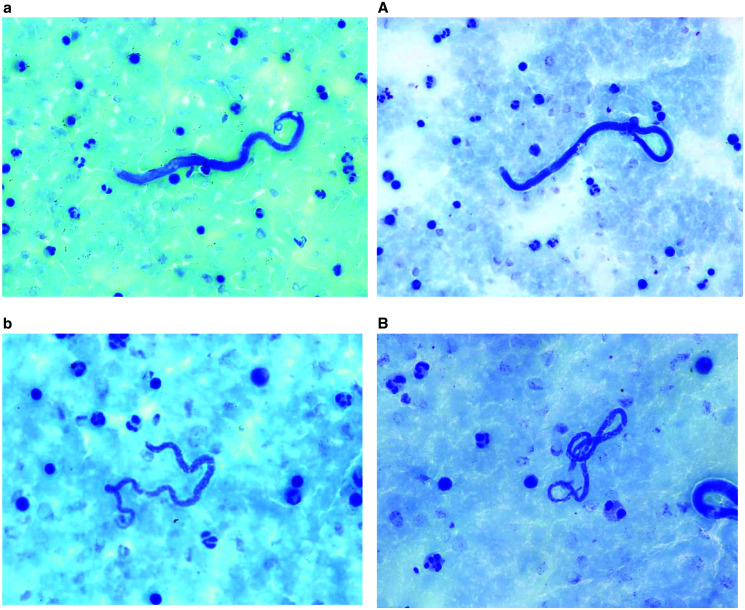
*Loa loa *and* Mansonella perstans *microfilariaemia in a blood smear stained with Field’s stain (**a** and **b**) and with Giemsa (**A** and **B**).

Considering only microfilaremic subjects, median *L. loa* microfilaraemia was 600 (IQR 200–1963) and 538 (IQR 200–1,463) microfilariae per ml blood for Giemsa-stained and Field’s stain treated samples, respectively. This resulted in a strong positive linear relationship (Spearman rank correlation coefficient ρ = 0.91, *P* < 0.001) (Table 2 and Figure 1). The relationship of both staining methods was only moderate positive for *M. perstans*, with 100 (IQR 100–200) mf/mL; median microfilaremia for both methods (Spearman rank correlation coefficient ρ = 0.54, *P* value = 0.002). The stained microfilariea parasites in smears appeared as shown in Figure 2.

**Table 2:** Number of microfilariae in Giemsa-stained and Field’s-stained samples.

	Quantitative assessment		
Species	Staining method	Median (IQR), Mf/mL	Spearman rank correlation coefficient ρ
Loa loa	Field	600 (200–1,962.5)	0.905, *P* < 0.001
	Giemsa	537.5 (200–1,462.5)
*Mansonella perstans*	Field	100 (100–200)	0.538, *P* = 0.002
	Giemsa	100 (100–200)

IQR = interquartile range; Mf/mL = microfilariae per milliliter.

## DISCUSSION

Microscopic diagnostics based on Field’s stain showed similar performance for *L. loa* detection and quantification compared with Giemsa staining. This high concordance with conventional Giemsa staining makes Field’s stain an attractive option for rapid diagnosis, which may be particularly useful for population-based screening programs. Importantly, microscopic screening for high microfilarial load as required for treatment decisions for loiasis or for exclusion of hypermicrofilaremic individuals in onchocerciasis controls programs using ivermectin mass drug administration, shows almost perfect concordance thus making Field’s stain a viable option in this setting.

In contrast to these results, the detection rate for *M. perstans* was lower for blood slides stained with Field’s stain compared with Giemsa staining. This finding may be explained by the shorter length and particularly the thinner diameter of *Mansonella* spp. compared with *L. loa*. Suboptimal staining of these structures may explain lower sensitivity and poorer correlation of quantification than for *L. loa* diagnostics.

Giemsa staining allows good visualization of the morphology of the microfilaria including the arrangement of nuclei, whereas the sheath is not ideally visualized by this conventional staining technique. Field’s stain similarly leads to good visualization of the morphology of microfilariae, allowing species differentiation by identification of the arrangement of nuclei and measuring of the length. Due to the short staining process, some microfilariae may be overstained or not sufficiently stained, the latter leading to a transparent appearance that may be overlooked. Similar to Giemsa staining, the sheath is not markedly visualized by this staining technique. Given the epidemiology of human filariasis in Gabon, where *L. loa* and *Mansonella* spp. occur, both staining techniques allow differentiation of microfilariae with certainty. A limitation that was observed for both staining techniques is when blood samples are not well fixed to the glass slide and parts of the thick blood smear, including microfilariae, are washed off during the staining and rinsing procedure. Finally, a subjective impression by the microscopists was that Field’s stain achieved higher quality staining results on freshly made thick blood smears compared with thick blood smears kept for several days before staining. This observation requires further systematic investigation.

This diagnostic study had some limitations, including the fact that patients were not randomly selected from the community but were selected for the presence of signs and symptoms suggestive for loiasis. Although the aim of this study was not to evaluate the prevalence of microfilarial infection, it should be noted that the prevalence of filariasis was high in this study cohort at ∼50%. However, this proportion is close to the estimated prevalence reported in the most recent population-based studies in the study area.^1^ It is possible that the probability of microfilaremia detection by light microscopy potentially depends not only on the type of staining method (Giemsa stain and Field’s stain) used for diagnostic purposes, but also on the order of slide sampling. However, slide staining does not take into account the order of sampling; therefore, potential bias may be equally distributed between staining methods. A strength of the study was that the microscopic analysis of slides was performed by blinding the microscopist to the identity of the patient and thus guaranteeing unbiased reading.

## CONCLUSIONS

In conclusion Field’s stain showed almost identical diagnostic performance to conventional Giemsa stain for *L. loa* parasites, for both qualitative and quantitative analysis. Considering the rapid staining time of less than 1 minute, Field’s stain may be considered as an alternative staining option particularly suitable for rapid microscopic detection of *L. loa* microfilariae in population-based screening programs.
